# Mutagenesis Reveals That the *OsPPa6* Gene Is Required for Enhancing the Alkaline Tolerance in Rice

**DOI:** 10.3389/fpls.2019.00759

**Published:** 2019-06-11

**Authors:** Bing Wang, Guoqiang Xie, Zhonglai Liu, Rui He, Jiao Han, Shengcai Huang, Laihua Liu, Xianguo Cheng

**Affiliations:** ^1^Laboratory of Plant Nutrition and Biology, Institute of Agricultural Resources and Regional Planning, Chinese Academy of Agricultural Sciences, Beijing, China; ^2^Department of Plant Nutrition, College of Resources and Environmental Sciences, China Agricultural University, Beijing, China; ^3^Jiujiang Academy of Agricultural Sciences, Jiujiang, China

**Keywords:** alkaline stress, CRISPR/Cas9, inorganic pyrophosphatase, metabonomics, mutagenesis, *OsPPa6* gene

## Abstract

Alkaline stress (AS) is one of the abiotic stressful factors limiting plant’s growth and development. Inorganic pyrophosphatase is usually involved in a variety of biological processes in plant in response to the abiotic stresses. Here, to clarify the responsive regulation of inorganic pyrophosphatase in rice under AS, the mutagenesis of the *OsPPa6* gene encoding an inorganic pyrophosphatase in rice cv. Kitaake (*Oryza sativa* L. ssp. japonica) was performed by the CRISPR/Cas9 system. Two homozygous independent mutants with cas9-free were obtained by continuously screening. qPCR reveals that the *OsPPa6* gene was significantly induced by AS, and the mutagenesis of the *OsPPa6* gene apparently delayed rice’s growth and development, especially under AS. Measurements demonstrate that the contents of pyrophosphate in the mutants were higher than those in the wild type under AS, however, the accumulation of inorganic phosphate, ATP, chlorophyll, sucrose, and starch in the mutants were decreased significantly, and the mutagenesis of the *OsPPa6* gene remarkably lowered the net photosynthetic rate of rice mutants, thus reducing the contents of soluble sugar and proline, but remarkably increasing MDA, osmotic potentials and Na^+^/K^+^ ratio in the mutants under AS. Metabonomics measurement shows that the mutants obviously down-regulated the accumulation of phosphorylcholine, choline, anthranilic acid, apigenin, coniferol and dodecanoic acid, but up-regulated the accumulation of L-valine, alpha-ketoglutarate, phenylpyruvate and L-phenylalanine under AS. This study suggests that the *OsPPa6* gene is an important osmotic regulatory factor in rice, and the gene-editing of CRISPR/Cas9-guided is an effective method evaluating the responsive regulation of the stress-induced gene, and simultaneously provides a scientific support for the application of the gene encoding a soluble inorganic pyrophosphatase in molecular breeding.

## Introduction

Soils with highly salinity-alkalinity are widely distributed in the world, and account for 10% of the arable land. Statistics shows that saline-alkaline soils of nearly 8.3 million ha are universally used for rice planting (Haefele et al., 2014). Alkaline stress (AS) usually hinders plant’s growth and development, leading to a decrease in biomass by triggering a series of physiological damage to the plants, and these damages are mainly demonstrated by affecting photosynthesis, respiration, enzymatic activity and accumulation profile of ions in plants (Yang et al., 2008; Bu et al., 2012). Particularly, AS not only alters the distribution of Cl^-^, NO_3_^-^, and H_2_PO_4_^-^ ions, but also exerts an influence on the balance of Na^+^ and K^+^ ions in plant cells (Chen et al., 2011). Study shows that most of the translocation and distribution of ions are closely associated with the H^+^-translocating inorganic pyrophosphatase (H^+^-PPase) widely presenting in plants (Davies et al., 1992; Tsai et al., 2014). Therefore, to better understand whether the soluble inorganic pyrophosphatase (sPPase) has a similar regulatory role in the transport and translocation of ions to the H^+^-PPase, evaluating the genes encoding sPPase is imperative to better exploit these genes in cultivating alkaline tolerant crop.

In plant kingdom, two typical inorganic pyrophosphatase (PPases), the H^+^-PPase and the sPPase have been identified to play important regulatory roles in response to abiotic stresses (Ferjani et al., 2011; Gutiérrez-Luna et al., 2018). Usually, the H^+^-PPase catalyzes a coupled reaction of PPi hydrolysis and active proton transport across membranes (Segami et al., 2014). Previous studies have found that the genes encoding the H^+^-PPase have positive responses to the abiotic stresses. For example, the transcripts of the *ZmVP1* gene in *Zoysia matrella* is significantly induced by salinity, drought and cold, and the overexpression of the *ZmVP1* gene in *Arabidopsis thaliana* reveals strong salt tolerance (Chen et al., 2015). Similarly, the *KfVP1* gene in *Kalidium foliatum* is induced by salt and drought, and the transgenic *A. thaliana* overexpressing the *KfVP1* gene enhances the salt and drought tolerance (Yao et al., 2012). Wang et al. (2016) reported that the *ScHP1* gene in rye is induced by cold, salt, drought and phosphate (P) starvation. Like the genes encoding the H^+^-PPase, the genes encoding the sPPase were also functionally explored in diverse plants. Study showed that both tobacco and potato overexpressing the gene encoding a sPPase of *E. coli* delays plant growth and demonstrated thicker leaves with discolored and small size compared to the wild type (Sonnewald, 1992). George et al. (2010) found that silencing the *psPPase* gene encoding a plastidial soluble inorganic pyrophosphatase in tobacco inhibits the tobacco growth, and alters the accumulation profiles of starch and chlorophyll, and affects carotenoid biosynthesis in tobacco leaves, thus limiting photosynthesis of tobacco and leading to a moderate wilting phenotype under drought stress. Hernández-Domíguez et al. (2012) confirmed that the transcripts of the *PvPPa1* gene encoding a sPPase are increased in the common bean leaves, and the transcripts of the *PvPPa4* are highly accumulated in both the oldest and the youngest leaves under P starvation. Moreover, May et al. (2011) found that the *AtPPsPase1* gene encoding a pyrophosphatase in the HAD superfamily reveals an optimal catalytic activity under alkalinity with higher pH, and that the *AtPPsPase1* gene is strongly induced and up-regulated by P starvation (Müller et al., 2007), indicating a direct participation of the PPases in phosphate metabolism in plant. While glutamine synthetase (GS2) in plant participates in nitrogen metabolism by in charge of ammonium (NH_4_^+^) fixation through a pathway of ATP-dependent (Kaminski et al., 2015). Hoshida et al. (2000) found that overexpressing the *GS2* gene demonstrates an enhanced increase in photorespiration capacity in the transgenic rice under salt stress. These reports show that alteration of photosynthesis system seems to have a closely association with the metabolism of nitrogen in plant (Fuentes et al., 2001; Oliveira et al., 2002). Therefore, investigating the transcript profile of the *OsGS2* gene under AS is beneficial to better understanding whether the physiological changes of the sPPase-mediated affect the GS2 activity.

Currently, a total of seven of putative isoforms of sPPase in rice are determined to exhibit similar protein structures to the sPPases in *Arabidopsis*, and six isoforms of sPPases share similarity in the predicted protein sequences, only the sPPase (Os02g0768600) protein demonstrates a lower similarity in sequences of amino acids, but has higher similarity with the AtPPa6 in *Arabidopsis*. Referring to the nomenclature of the gene encoding sPPase in *Arabidopsis* (Schulze et al., 2004), the sPPase (Os02g0768600) in rice was named as the OsPPa6. Multiple alignment shows that the *OsPPa6* gene (accession: AK059725) in rice shares highly homology with the *ThPP1* gene (accession: KC250018) in *Thellungiella halophila*, and a previous report showed that the *ThPP1* gene, encodes a sPPase in *T. halophila*, is induced by AS, and obviously confers an enhanced alkaline tolerance in the transgenic rice (He et al., 2017).

At present, the genes encoding the sPPase were mainly characterized by overexpressing or silencing the target gene of RNAi-mediated in the plant. However, the regulatory role of the gene encoding sPPase in rice is not complete clear under AS. Particularly, the mutagenesis of the gene encoding the sPPase has not been reported yet. In this study, we used the CRISPR/Cas9 gene-editing system performed the mutagenesis of the *OsPPa6* gene in rice *Oryza sativa* japonica L. cv. Kitaake, and obtained two independent mutants of T_3_ generation with base mutation or insertion. To identify the regulatory role of the *OsPPa6* gene in response to AS, both the rice mutants and the wild type were subjected to AS, and investigated by profiling the physiological changes and the metabonomics-based metabolites differences. We are expected that manipulating the CRISPR/Cas9 gene-editing system not only is an effective pathway in identifying the functional genes, but also provides a scientific evidence for the application of the alkali-induced *OsPPa6* gene.

## Materials and Methods

### Vector Construction and Transformants Generation

A specific target fragment of 20-bp at the upstream of the PAM (NGG) motif in the cDNA of the *OsPPa6* gene (accession: AK059725) was identified by the Cas-OFFinder^1^ to avoid occurrence of the off-target effect. A pair of specific primers for the *OsPPa6* gene-editing were designed by the online guide design tool^2^, and connected by the two adapters, 5′-GGCA-3′ at the 5′ terminus and 5′-AAAC-3′ at the 3′ terminus, respectively (sgRNA-F/R, Supplementary Table S1). The annealing reaction was performed in a 20-μL of reaction solution containing 1 μL of annealing buffer, 4.5 μL of 100 μM of Editing-F/R, respectively, and 10 μL of ddH_2_O to form sgRNA fragments with complementary base pairing. The reaction procedure was performed by culturing at 95°C for 5 min, then following a gradient culture from 95°C to 70°C, which is decreased by 1°C in 1 min, and stored at 10°C. The pCXUN-U3 vector used for gene-editing was digested with *Aar*I at 37°C for 3–6 h, and the generated vector fragments were recovered and sequenced. The sgRNA fragments were ligated with the vector fragments in a reaction solution containing 1 μL of T4 ligase buffer, 2 μL of pCXUN-U3 vector, 3 μL of 10 μM of sgRNA, 0.5 μL of T4 ligase (5U μL^-1^) and 3.5 μL of ddH_2_O, then thoroughly mixed and incubated at 25°C over 10 min, and the fusion vector with sgRNA was transformed into the *E. coli* DH5α, and the generating transformants were cultured in a culture solution of 500 μL LB medium lacking antibiotics with 200 rpm at 37°C for 1 h. Then, total 100 μL of the culture medium were evenly spread on the LB plate containing Kan and cultured at 37°C under darkness overnight. One independent clone was chosen to further culture and detected by PCR using the universal primers U3-F/R (Supplementary Table S1). The plasmid DNA of transformants was extracted, and subsequently transferred into the *A. tumefaciens* competent cells of EHA105, and the generated transformants were cultured in a culture solution of 500 μL liquid medium lacking antibiotics with 230 rpm at 28°C overnight. A total of 100 μL of the culture solution was evenly spread on the LB plate containing Kan and rifampicin and cultured at 28°C under darkness overnight. Finally, a single plaque was picked up and cultured by the same condition, the generating bacterial culture solution was used for PCR detection using the specific primers OsPPa6-F/R (Supplementary Table S1), and the PCR products were sequenced to determine the positive strains.

### Transformation and Screening of Mutant Plants

The transformants of *A. tumefaciens* EHA105 strains were transferred into immature embryo calli of rice cv. Kitaake (*O. sativa, L. japonica*). After two rounds of screening on the N_6_ solid medium (Chu et al., 1975) containing 2,4-D (2,4-Dichlorophenoxyacetic acid) and hygromycin (Nishimura et al., 2006), the infected calli were cultured on the N_6_ solid medium lacking 2,4-D under darkness of 8 h and photolight of 16 h with a 70% humidity at 26°C for 1 month for generation of the adventitious buds, then the calli with adventitious buds were further cultured on the ½ MS solid medium for 1 month to induce roots regeneration. Regenerated seedlings were further cultured in peat soils to harvest the seeds of T_0_ generation under natural condition. The mutants of T_1_ generation were generated by self-bred of T_0_ generation seeds. The leaf genomic DNA of the mutants of T_1_ generation was extracted and used for PCR detection by the specific primers OsPPa6-F/R and Cas9 primers Cas9-F/R (Supplementary Table S1). Both the line 9 and line 13 were confirmed to be two positive homozygous mutants with cas9-free, and the mutant seeds of T_3_ generation were obtained by continuously screening under the same culture condition.

### Plant Culture and Stress Treatment

Rice seeds were germinated on moist filter paper at 30°C under darkness. A total of 50 of grains from each line (WT and mutants) were cultured to investigate the germination rates, and the generating seedlings were transplanted into the mixture of peat soil and vermiculite (2:1) for further culture in a greenhouse with a humidity of 60%∼70% at 28°C under a 16 h light intensity of 350∼400 μmol m^-2^ s^-1^ and at 22°C for 8 h in the dark (Thimijan and Royal, 1983). The seedlings with 3∼4 leaves were subjected to AS (AS, 50 mM, molar ratio of NaHCO_3_/Na_2_CO_3_ 9:1; pH 9.17) (Montesinos-Pereira et al., 2018) for 2 weeks, then the seedlings showing phenotype differences were sampled for physiological measurements and molecular detection, and the remained seedlings were continuously cultured to investigate rice grain yield of per plant at mature stage. Plant samples were collected at required time points and used for further analyses or total RNA extraction.

### Isolation of Nucleotides and Quantitative RT-PCR

Genomic DNA of rice leaves was prepared by the method of CTAB (Allen et al., 2006). Total RNA of leaves was extracted by Easy Pure Plant RNA Kit (*Trans* Gene Biotech, Beijing, China). Quantitative RT-PCR procedures were carried out according to the manufacturer’s instructions by the Light Cycler System (Bio-Rad, Richmond, CA, United States) with the SYBR^^®^^ qPCR Master Mix Kit (*Trans* Gene Biotech, Beijing, China) by a pair of primers (Supplementary Table S1). The relative expression levels were calculated by the 2^-ΔΔCT^ formula (Livak and Schmittgen, 2001) using the reference gene (accession: XM_015774830) as an internal standard.

### Physiological Measurements

The contents of phosphate, Na^+^ and K^+^ were determined as described in the following procedures, respectively. Approximately, 250 mg of dried matter were digested by 5 mL of the concentrated sulfuric acid and 2 mL of 30% H_2_O_2_, and diluted to a volume of 100 mL in a volumetric flask by ultrapure water. The phosphate was determined by the vanadate–molybdate-yellow colorimetric method using a Continuous Flowing Analyzer (SEAL Analytical AA3, Germany), and the contents of Na^+^ and K^+^ were determined by atomic absorption (ZEEnit^^®^^700P, Germany). The ATP contents in plant tissues were extracted by ddH_2_O and measured by the method described previously with HPLC system (Agilent 1290 Infinity II, United States) (Surova et al., 2016). Pyrophosphate was extracted by a previous method (Dancer and Rees, 1989) and determined by the oxidation of NADH at 340 nm using a spectrophotometer (Thermo Scientific GENESYS10S, United States) (Smyth and Black, 1984). The photosynthetic parameters of the first flag leaf of rice were measured by a portable photosynthesis measurer Li-6400XT (Li-COR, United States). The chlorophyll completely was extracted by 80% acetone (Lichtenthaler, 1987) and determined at 645 nm and 663 nm by spectrophotometer, respectively, and calculated by a formula of *C* (mg g^-1^ FW) = (20.29 × OD_645_ + 8.05 × OD_663_) × V × 10^-3^/FW, where FW means fresh weight and V means extract volume (Croft et al., 2017). The D (+)-sucrose was extracted by a solution of 4 mL of 80% ethanol and determined at 480 nm using a spectrophotometer by m-dihydroxybenzene method (Hendrix, 1993). Both the starch and soluble sugar in rice were quantitatively measured at 620 nm by the anthrone-sulfuric assay using a spectrophotometer (Yemm and Willis, 1954). The content of free proline was determined as the described method by Bates et al., 1973 in. The malondialdehyde (MDA) was extracted by a previous method (Hodges et al., 1999), and separately measured at 450, 532, and 600 nm and calculated by a formula of *C* (μ*mol g*^-1^ *FW*) = [6.45 × (OD_532_ - OD_660_) - 0.56 × OD_450_]/(FW × 1000) (Khan et al., 2017). The osmotic potentials of leaves were measured using an osmotic pressure dew point meter (Wescor 5520, Logan, UT, United States) by a formula of Ψ*s* (MPa) = -C_i_ × 0.008314 × (273+T) × 10^-3^, where C_i_ means instrument reading and T represents environment temperature (Ball and Oosterhuis, 2005).

### Metabolites Extraction and LC-MS/MS Analysis

The metabolites of rice were extracted and analyzed by the method described previously (Zhang Y. et al., 2017). Briefly, a total of 50 mg of fresh leaves was ground in liquid nitrogen, and transferred into the new EP tube containing 1 mL of an internal target substance (V_methanol_:V_acetonitrile_:V_water_ = 2:2:1, which was kept at -20°C in advance), and homogenized by ball mill at 45 Hz for 4 min, then treated on ice bath by continuous three times of ultrasound for 5 min, then incubated at -20°C for 1 h to precipitate proteins, then centrifuged by 14500 × *g* at 4°C for 15 min. A total of 500 μL of supernatants were transferred into the new EP tubes, and dried in a vacuum concentrator without heating, and was supplemented by a reconstitution with 100 μL of extraction solution (V_acetonitrile_:V_water_ = 1:1), and mixed for 30 sec, and sonicated in a water bath at 4°C for 10 min, and centrifuged by 14500 × *g* at 4°C for 15 min. The supernatants were filtered through the PTFE membrane of 0.22 mm (Sigma, United States), and subjected to analyses on the UHPLC-QTOF-MS (1290, Agilent Technologies) equipped with a UPLC BEH Amide column (1.7 μm, 2.1 mm × 100 mm, Waters Corporation, United States) using optimized mobile phases. The extraction and analysis of metabolites were assisted by the Allwegene Technology Co., Ltd. (Beijing, China).

### Statistics Analysis

MS data preprocessing such as alignment, peak intensity, retention time (RT) correction and mass-to-charge ratio (m/z) values was performed using Markerlynx XS^TM^ software (Waters Corporation, United States). Orthogonal projection to latent structures-discriminant analysis (OPLS-DA) was applied to the supervised multivariate analysis using SIMCA-P software (version 12.0). The supervised OPLS-DA was used to reconstruct the metabonomics data and scientifically evaluate the differential variables expressions of separation by reasonably amplifying differences between the experimental and control groups (Zhang Y. et al., 2017). Moreover, a completely randomized design involving an arrangement of 3 (WT and two mutants) × 2 (control and AS) factor was established in this experiment. Univariate analyses were statistically performed by using one-way ANOVA in the statistical software SPSS 17.0 (SPSS, Chicago, IL, United States). Based on the results of ANOVA, a DUNCAN-test was analyzed. All data were represented by an average with a standard error of three replicates. The significance level was expressed by *P* ≤ 0.05 or 0.01.

## Results

### *OsPPa6* Is a Member of sPPase Encoding a Soluble Inorganic Pyrophosphatase

**FIGURE 1:**
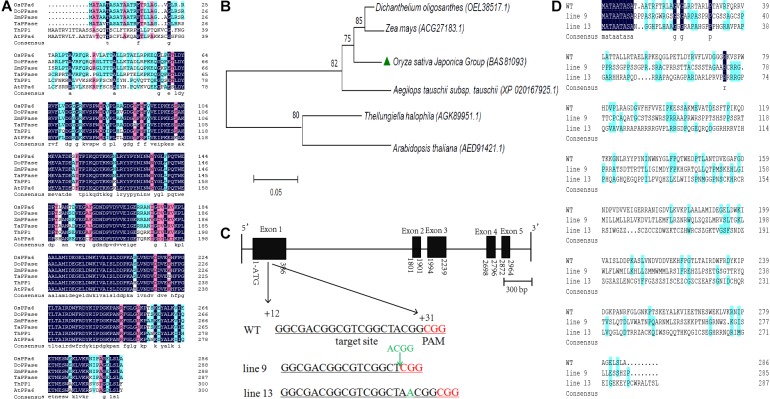
Bioinformatics analyses of the soluble inorganic pyrophosphatase and mutagenesis identification of the *OsPPa6* gene. **(A)** Homology alignment of soluble inorganic pyrophosphatase. OsPPa6, *O. sativa* Japonica Group (accession: BAS81093); DoPPase, *Dichanthelium oligosanthes* (accession: OEL38517); ZmPPase, *Zea mays* (accession: ACG27183); TaPPase, *Aegilops tauschii* (accession: XP_020167925); ThPP1, *Thellungiella halophila* (accession: AGK89951); AtPPa6, *Arabidopsis thaliana* (accession: AED91421). Blue and pink color indicate the similarity; white color indicates the differences; **(B)** Phylogenetic tree analysis of the soluble inorganic pyrophosphatase. **(C)** Structural profiles of the *OsPPa6* gene with the mutagenesis of the CRISPR/Cas9-mediated in the first exon. Black rectangle means the exon. Black line means the intron. The underlined nucleotides indicate the target sites. Nucleotides in red represent PAM sequences. PAM, protospacer adjacent motif. The target sequences are located at +12th to +31th of the gene; **(D)** Multiple alignments of the OsPPa6 proteins between the wild type and mutants of CRISPR/Cas9-edited.

The *OsPPa6* gene encodes a protein of 286 amino acid and shares more than 90% of homology with the genes encoding the sPPase in *Dichanthelium oligosanthes, Zea mays*, and *Aegilops tauschii*. The OsPPa6 protein also shares homology of 71.2% with the ThPP1 in *T. halophila* and 68.0% with the AtPPa6 in *A. thaliana* (Figure 1A). Bioinformatics analysis shows that the OsPPa6 has a closely familiarity with the DoPPase and ZmPPase in diverse plants (Figure 1B). Moreover, a total of six of putative protein encoding the sPPase in rice were retrieved by the Rice Data database^3^ and are represented by Os01g0974800, Os01g0866500, Os02g0704900, Os04g0687100, Os05g0114000, and Os05g0438500, respectively. These six proteins share more than 75% of homogeneity, however, the OsPPa6 (Os02g0768600) only shares less than 30% of homology with these six putative proteins (Supplementary Figure S1), indicating that the *OsPPa6* gene exhibits structural specificity in the sPPase family.

### The Mutagenesis of Nucleotides in the *OsPPa6* Gene

Firstly, based on the specific recognition of the CRISPR/Cas9 gene editing system, the target sites in the clustered regularly interspaced short palindromic repeat sequences of the coding region of the *OsPPa6* gene are determined, and located at the fragment between the +12th and +31th in the coding region (Figure 1C). PCR detection was continuously performed to confirm the mutagenesis of the target gene in the rice mutant. PCR products sequencing shows that four bases (A, C, G, G) at the target site in the line 9 were deleted and an A base was inserted into the +18th site of the target sequence in the line 13 compared with the WT (Figure 1C and Supplementary Figure S1), thus altering the sequences of amino acids in the OsPPa6 protein in the mutant lines (Figure 1D). Therefore, the encoded amino acids sequences in both line 9 and line 13 were obviously interfered by the deletion or insertion of a single base, thus triggering a loss of function of the *OsPPa6* gene in rice. Homology analysis shows that the OsPPa6 shares lower similarity with the other six putative proteins of sPPase in rice, and exhibits obvious differences and specificity in nucleotide sequences of the CRISPR/Cas9 recognition target site compared to the other putative genes encoding the sPPase in rice (Supplementary Figure S2). PCR and sequencing also confirmed that no off-target effects were observed in the mutants, indicating that the other six putative genes encoding the sPPase were not edited by the CRISPR /Cas9 system.

### The Mutagenesis of the *OsPPa6* Gene Delayed Rice Growth

**FIGURE 2:**
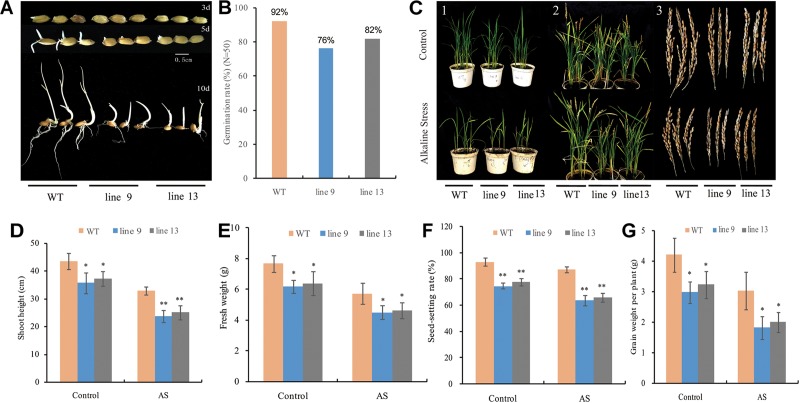
Changes in phenotype and biomass of rice under favorable condition or alkaline stress (AS). **(A)** Germination time; **(B)** Germination rate; **(C)** (1) Rice phenotype at seeding stage; (2) Rice phenotype at maturity stage; and (3) Seed differences in phenotype; **(D)** Shoot height of the seedlings; **(E)** Fresh weight of the seedlings; **(F)** Seed-setting rate; and **(G)** Grain weight of per plant. Values are mean ± SD of six replications and asterisks denote Student’s test significance at level of *P* ≤ 0.05 (^∗^) or *P* ≤ 0.01 (^∗∗^).

**FIGURE 3:**
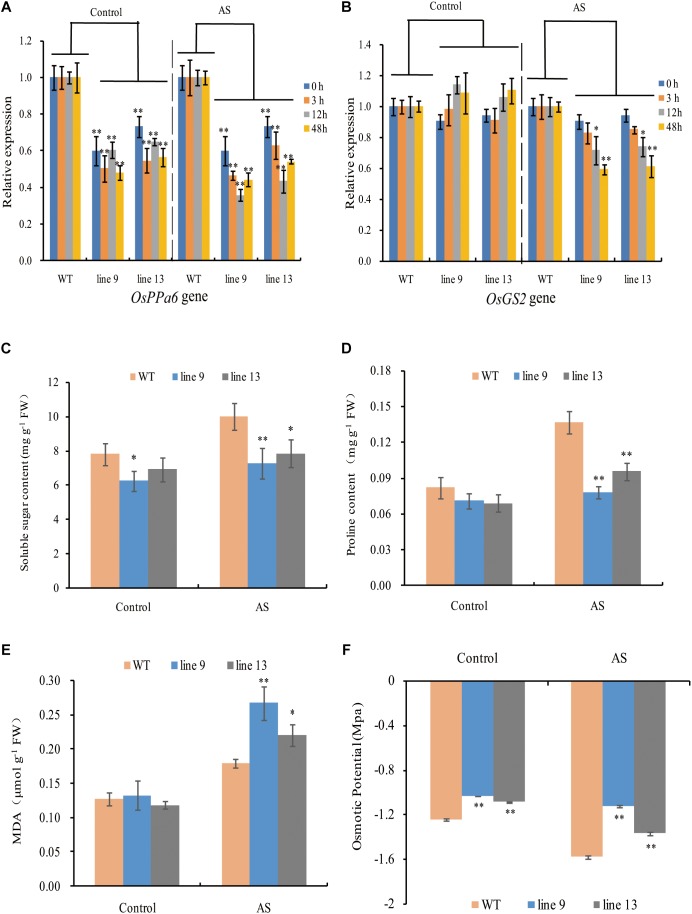
Gene relative expression levels and physiological changes under favorable culture or AS. **(A)** Relative expression levels of the *OsPPa6* gene in rice shoots; **(B)** Relative expression levels of the *OsGS2* gene in rice shoots; **(C)** Soluble sugar content; **(D)** Proline content; **(E)** MDA content; **(F)** Osmotic potential. Values are mean ± SD of three replications and asterisks denote Student’s test significance at level of *P* ≤ 0.05 (^∗^) or *P* ≤ 0.01 (^∗∗^).

To observe the effect of the mutagenesis of the *OsPPa6* gene on rice growth, we investigated rice germination rates in a growth chamber under favorable culture and performed pot experiment in a greenhouse under AS as described in the method. Data showed that both the line 9 and line 13 delayed germination time under favorable culture condition compared to the WT (Figure 2A). After 10-day of culture, both the line 9 and line 13 only showed 76 and 82% of germination rates, respectively, while the WT demonstrated 92% of germination rates (Figure 2B), suggesting that the mutagenesis of the *OsPPa6* gene affected the seed germination. To investigate the growth change of rice in response to AS, both the mutants and the WT seedlings with 3∼4 leaves were cultured under AS for 2 weeks, and the line 9 and line 13 obviously showed a growth inhibition compared with the WT under both favorable culture and AS stress (Figure 2C-1). Investigation showed that the average plant heights of the line 9 and the line 13 were 35.64 cm and 37.16 cm, respectively, which significantly were lower than 43.45 cm of the WT at the seeding stage even at favorable culture. In the case of culture under AS, both the line 9 and line 13 revealed lower growth rates, their average plant heights were only 23.75 cm and 25.04 cm, respectively, and demonstrated obvious dwarf phenotype compared with the WT (Figure 2D). Similarly, the fresh weights of the line 9 and line 13 were decreased by 19.32 and 16.58% compared with the WT under favorable culture, respectively. In the case of the culture under AS, the line 9 and line 13 demonstrated a decrease of 21.29 and 19.20% in the fresh weights of per plant compared with the WT, respectively (Figure 2E). At the maturity stage, the two mutants still revealed a lower plumpness of grains compared to the WT even at favorable culture condition (Figure 2C-2,3), and that the seed-setting rates of 74.77% in the line 9 and 77.78% in the line 13 were significantly lower than the seed-setting rates of 93.22% in the WT. In accordance with favorable culture, the seed-setting rates of 63.82% in the line 9 and 66.07% in the line 13 were significantly lower than the seed-setting rates of 87.30% in the WT under AS (Figure 2F). Moreover, the grain yields of the line 9 and line 13 of per plant were decreased by 1.22 g and 0.97 g compared to the WT under favorable culture, respectively, and that the line 9 and line 13 led to a decrease of 40.26 and 33.99% in the grain yield of per plant compared with the WT under AS, respectively (Figure 2G).

### Transcripts of *OsPPa6* Gene and *OsGS2* Gene

To profile the expression of the *OsPPa6* gene in the WT, we performed analyses of qRT-PCR using the total RNA in leaves. The data showed that the *OsPPa6* gene in the WT was significantly induced and its transcripts were increased by 1.81-fold after 12-h of AS relative to the transcripts in the treatment without AS (Supplementary Figure S3). Moreover, the expression levels of the *OsPPa6* gene in the mutants were significantly lower than that in the WT, especially under AS (Figure 3A), suggesting that the mutagenesis of the *OsPPa6* gene reduced the transcripts of the *OsPPa6* gene. Unlike the *OsPPa6* gene, the*OsGS2* gene encoding a chloroplastic GS2 in the WT was significantly inhibited after 48 h of AS, and its relative expression levels lowered 35% compared to the measured value under the favorable culture (Supplementary Figure S3), suggesting that the *OsGS2* was not induced by AS. Accordingly, as shown in Figure 3B, both the mutants and WT had no significant differences in the transcripts of the *OsGS2* gene under favorable culture, but the transcript levels of the *OsGS2* gene in the mutants were significantly lower than that in the WT with the prolongation of AS, indicating that the *OsPPa6* gene and the *OsGS2* gene demonstrated differential expression profiles in rice under AS.

### A Loss of Function of the *OsPPa6* Gene Lowered the Alkaline Tolerance in Rice

To characterize the physiological responses of the mutants and the WT to AS, we investigated the accumulation profiles of physiological compatible metabolites and performed photosynthetic measurements. Compatible metabolites are not only involved in the regulation of osmotic potentials, but also provide energy resource for the metabolism in plant, and thereby affecting the process of photosynthesis, conversely altering the accumulation of compatible metabolites such as soluble sugars and proline. Therefore, we measured inorganic phosphate (P), ATP, PPi, soluble sugar, proline, MDA, osmotic potential, photosynthesis, chlorophyll, sucrose, starch, Na^+^ and K^+^ in rice to evaluate the effects of the mutagenesis the *OsPPa6* gene on accumulation of metabolites and photosynthetic process in response to AS. Measurements showed that when the rice were subjected to AS, the line 9 and line 13 led to a decrease of 0.30 mg g^-1^ and 0.23 mg g^-1^ in P content, and decrease of 19.63 and 17.68% in the ATP content compared to the WT, respectively, however, the line 9 and line 13 demonstrated an increase of 44.67 and 39.21% in the PPi content compared with that in the WT, respectively (Table 1). Data also showed that both the line 9 and line 13 lowered the contents of soluble sugar and proline than the WT under AS, and showed a decrease of 27.13 and 21.32% in the contents of soluble sugars, respectively (Figure 3C), and a decrease of 42.86 and 28.57% in the contents of proline compared with the WT, respectively (Figure 3D). However, in the case of AS, both the line 9 and line 13 obviously increased the accumulation of the MDA and the osmotic potentials in leaves, and showed an increase of 0.50- and 0.22-fold in the MDA contents compared to the WT, and an increase of 0.45 MPa and 0.21 MPa in the osmotic potentials of leaves compared with the WT, respectively (Figure 3E,F).

**Table 1:** The contents of inorganic phosphorus, ATP and pyrophosphate.

Lines	inorganic phosphorus (P) (mg g^-1^)		ATP (nmol g^-1^)		Pyrophosphate (PPi) (nmol g^-1^)	
			
	Control	As	Control	As	Control	As
WT	2.89 ± 0.20 a	1.61 ± 0.11 a	131.66 ± 7.00 a	94.91 ± 6.85 a	17.69 ± 1.63 b	15.94 ± 1.58 b
line 9	1.85 ± 0.07 b	1.31 ± 0.09 b	111.38 ± 6.78 b	76.28 ± 5.74 b	25.87 ± 1.10 a	23.06 ± 1.84 a
line 13	1.90 ± 0.12 b	1.38 ± 0.04 b	115.49 ± 6.19 a	78.13 ± 4.41 b	23.77 ± 1.56 a	22.19 ± 1.42 a


*Values are the mean ± SD (*n* = 3). Different letters indicate that mean values are significantly different between WT and the gene-edited lines (*P* ≤ 0.05).*

Photosynthetic measurements showed that the mutagenesis of the *OsPPa6* gene significantly lowered the net photosynthetic rate (P_n_) in the mutant lines under favorable treatment. Especially, the line 9 not only showed a decrease of 0.49 μmol m^-2^ s^-1^ in P_n_, but also demonstrated a decrease of 0.057 mol m^-2^ s^-1^ in stomatal conductance (G_s_), and the intercellular carbon dioxide concentration (C_i_) in the line 13 was also significantly lower than that in the WT under favorable condition (Table 2). In the case of AS, the line 9 and line 13 lowered 22.62 and 20.03% of the P_n_, and 16.88, and 12.81% of the G_s_ compared to the WT, respectively. Meanwhile, the C_i_ of the line 9 and line 13 were decreased by 12.96 μmol mol^-1^ and 17.34 μmol mol^-1^, and the transpiration rates (T_r_) of the line 9 and line 13 were decreased by 18.65 and 12.97% compared to the WT under AS, respectively (Table 2). These data indicate that the mutagenesis of the *OsPPa6* gene resulted in a remarkable decrease in photosynthetic capacity in the mutants compared with the WT under AS. In photosynthesis, the chlorophyll functions in the absorption and transformation of light energy. Therefore, we investigated the accumulation responses of the chlorophyll in the mutants because of the mutagenesis of the *OsPPa6* gene under AS. Data show that no significant differences in the chlorophyll accumulation were observed between the mutants and the WT under favorable culture, but, in the case of culture under AS, the line 9 and line 13 significantly triggered a decrease of 0.67 mg g^-1^ and 0.5 mg g^-1^ of the chlorophyll compared to the WT, respectively (Table 3). Measurements also exhibit that chlorophyll accumulation and photosynthetic capacity affected the accumulation of sucrose and starch metabolites from plant photosynthesis because the accumulation of sucrose and starch in the mutants were significantly lowered compared with the WT under AS. The sucrose of 1.85 mg g^-1^ in the line 9 and 1.78 mg g^-1^ in the line 13 are significantly lower than the sucrose of 2.53 mg g^-1^ in the WT under AS, and the starch contents in the line 9 and line 13 were also decreased by 36.17 and 27.66% compared to the WT, respectively (Table 3). These results suggest that mutants obviously lowered the photosynthetic capacity and chlorophyll contents, consequently reducing the accumulation of sucrose and starch under AS.

**Table 2:** Photosynthetic parameters of rice leaves.

Treatments	Lines	P_n_	G_s_	C_i_	T_r_	WUE
						
		μmol m^-2^ s^-1^	mol m^-2^ s^-1^	μmol mol^-1^	mmol m^-2^ s^-1^	μmol mmol^-1^
Control	WT	12.32 ± 0.06 a	0.373 ± 0.014 a	166.29 ± 2.29 a	4.17 ± 0.42 a	2.97 ± 0.29 a
	line 9	11.83 ± 0.07 c	0.316 ± 0.018 b	163.72 ± 1.93 ab	4.03 ± 0.35 a	2.95 ± 0.24 a
	line 13	12.01 ± 0.06 b	0.394 ± 0.022 a	160.45 ± 2.02 b	4.15 ± 0.13 a	2.90 ± 0.076 a
AS	WT	7.34 ± 0.24 a	0.320 ± 0.016 a	137.86 ± 1.05 a	3.70 ± 0.14 a	1.98 ± 0.01 a
	line 9	5.68 ± 0.11b	0.266 ± 0.021 b	124.90 ± 1.33 b	3.01 ± 0.26 b	1.89 ± 0.13 ab
	line 13	5.87 ± 0.13 b	0.279 ± 0.019 b	120.52 ± 5.14 b	3.22 ± 0.11 b	1.82 ± 0.02 b


*Values are the mean ± SD (*n* = 3). Different letters indicate that mean values are significantly different between WT and the mutant lines (*P* ≤ 0.05). *P_*n*_*, net photosynthetic rate; *G_*s*_*, stomatal conductance; *C_*i*_*, intercellular carbon dioxide concentration; *T_*r*_*, transpiration rate; and *WUE*, water use efficiency.*

**Table 3:** The contents of chlorophyll, sucrose and starch.

Lines	Chlorophyll (mg g^-1^ FW)		Sucrose (mg g^-1^ FW)		Starch (mg g^-1^ FW)	
			
	Control	As	Control	As	Control	As
WT	4.02 ± 0.11 a	3.08 ± 0.21 a	3.59 ± 0.31 a	2.53 ± 0.21 a	0.62 ± 0.034 a	0.47 ± 0.029 a
line 9	3.83 ± 0.13 a	2.41 ± 0.14 b	3.46 ± 0.33 a	1.85 ± 0.31 b	0.64 ± 0.062 a	0.30 ± 0.048 b
line 13	3.77 ± 0.15 a	2.58 ± 0.28 b	3.94 ± 0.21 a	1.78 ± 0.28 b	0.68 ± 0.046 a	0.34 ± 0.036 b


*Values are the mean ± SD (*n* = 3). Different letters indicate that mean values are significantly different between WT and the gene-edited lines (*P* ≤ 0.05).*

Except for the metabolites accumulation and photosynthesis, we also measured the contents of Na^+^ and K^+^ in rice leaves to investigate whether the mutagenesis of the *OsPPa6* gene influence the distribution of Na^+^ and K^+^ under AS. Our data show that the mutagenesis of the *OsPPa6* gene significantly affected the accumulation of Na^+^ and K^+^ in the mutants. Especially, when rice was exposed to AS, the mutants demonstrated an increase of 14.42% in line 9 and 11.70% in line 13 in the Na^+^ contents compared with the WT, but lowered 7.39% in line 9 and 3.60% in line 13 in the K^+^ contents compared with the WT, respectively, thus leading to an increase of 23.61% in Na^+^/K^+^ ratio in the line 9 and 15.28% in the line 13 comparing to the WT, respectively (Table 4). These data indicate that the mutagenesis of the *OsPPa6* gene increased accumulation of Na^+^ and reduced the accumulation of K^+^ in leaves of the mutants, and lowered the alkaline tolerance because of the damage from excess uptake of Na^+^ in plant cells.

**Table 4:** The contents of Na^+^, K^+^, and Na^+^/K^+^ ratio.

Lines	Na^+^ content (mg g^-1^ DW)		K^+^ content (mg g^-1^ DW)		Na^+^/K^+^ Ratio	
			
	Control	As	Control	As	Control	As
WT	2.66 ± 0.04 a	14.36 ± 0.41 b	17.33 ± 0.46 a	10.01 ± 0.50 a	0.15 ± 0.003 a	1.44 ± 0.041 b
line 9	2.51 ± 0.07 a	16.43 ± 0.70 a	16.16 ± 0.98 ab	9.27 ± 0.54 a	0.16 ± 0.014 a	1.78 ± 0.163 a
line 13	2.55 ± 0.08 a	16.04 ± 0.82 a	15.84 ± 0.51 b	9.65 ± 0.30 a	0.16 ± 0.004 a	1.66 ± 0.088 a


*Values are the mean ± SD (*n* = 3). Different letters indicate that mean values are significantly different between WT and the gene-edited lines (*P* ≤ 0.05).*

### The Mutagenesis of the *OsPPa6* Gene Revealed Metabolic Differences in Rice

**FIGURE 4:**
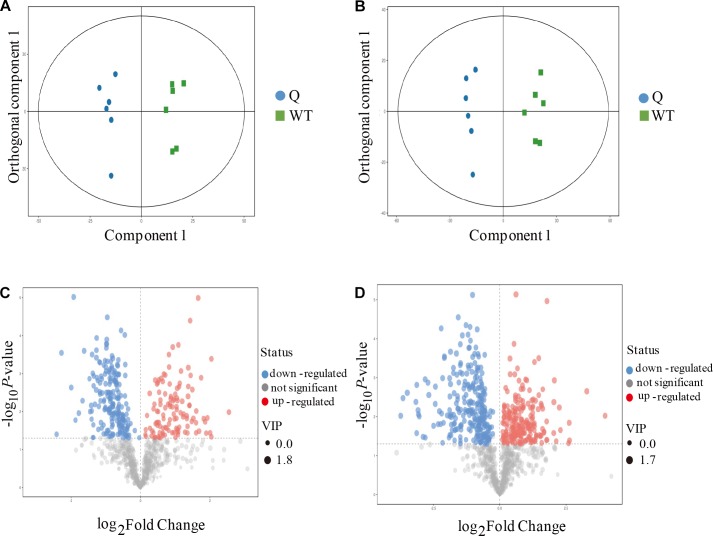
Metabolomics analyses of the mutants and the wild type. **(A)** Score scatter plot of OPLS-DA model for group Q vs WT (positive ion mode); **(B)** Score scatter plot of OPLS-DA model for group Q vs WT (negative ion mode); **(C)** Volcano plot for group Q vs WT (positive ion mode); **(D)** Volcano plot for group Q vs WT (negative ion mode).

To better explore the differential metabolites between the mutants and the WT under AS, the mutant line 9 (experimental group) and the WT (control group) were analyzed by UPLC-MS to obtain a higher level of group separation and understand the metabolic differences triggered by the mutagenesis of the *OsPPa6* gene. Our data show that the parameters of the OPLS-DA for the classification were expressed by the R^2^Y (cum) of 0.975 and Q^2^ (cum) of 0.782 representing the positive ion mode, and by the R^2^Y(cum) of 0.978 and Q^2^(cum) of 0.878 representing the negative ion mode, respectively. These parameters representing the two ion models exhibited a better stability and predictability, and effectively reflected the metabolic differences between the WT and the mutant in response to AS. The separation trends in both the experimental group and the control group were remarkable under positive or negative ion model (Figure 4A,B). The detection demonstrates that the mutagenesis of the *OsPPa6* gene obviously altered the distribution of the metabolites in the mutant under AS. As shown in Figure 4C,D, the volcano plots expressing the differential metabolites revealed significant differences between the experimental and the control groups. Using VIP screening (variable importance in the projection) >1 at a level of *P* < 0.05, a total of 324 of the differential metabolites were isolated in the positive ion mode. In detail, the experimental group revealed a total of 117 of the up-regulated metabolites (red dot, Figure 4C) and a total of 207 of the down-regulated metabolites (blue dot, Figure 4C) relative to the control group. In the same way, a total of 482 of the differential metabolites were screened in the negative ion mode, in which a total of 215 of the differential metabolites were up-regulated (red dots, Figure 4D) and a total of 267 of the differential metabolites were down-regulated (blue dots, Figure 4D) in the experimental group compared with the control group, respectively. Furthermore, using screening conditions with more than 1 of VIP and fold change at a level of P less than 0.05, we isolated out a total of 10 of the differential metabolites that are mainly involved in different metabolic pathways. Analyses show that the mutagenesis of the *OsPPa6* gene not only obviously down-regulated accumulation of phosphorylcholine, choline, anthranilic acid, apigenin, coniferol, and dodecanoic acid, but also up-regulated the accumulation of L-valine, alpha-ketoglutarate, phenylpyruvate, and L-phenylalanine in the mutants under AS (Table 5).

**Table 5:** Identification of partial differential metabolites.

	Retention time/min	m/z[M]^+^	Formula	Identification	Relative Quantitative Mean Q/(WT)	Up/down	Pathway
Positive-ion	470.62	184.07	C_5_H_14_NO_4_P	phosphorylcholine	0.18 (0.29)	down	Glycerophospholipid metabolism
	253.32	104.11	C_5_H_14_NO	choline	1.80 (2.00)	down	Glycerophospholipid metabolism
	267.53	275.10	C_7_H_7_NO_2_	anthranilic acid	0.02 (0.05)	down	Tryptophan metabolism
	41.89	331.08	C_15_H_10_O_5_	apigenin	1.92 (4.00)	down	Flavone and flavonol biosynthesis
	236.92	399.13	C_10_H_12_O_3_	coniferol	0.17 (0.24)	down	Biosynthesis of phenylpropanoids
	80.24	218.21	C_12_H_24_O_2_	dodecanoic acid	0.07 (0.11)	down	Fatty acid biosynthesis
Negative-ion	280.28	116.07	C_5_H_11_NO_2_	L-valine	0.11 (0.04)	up	Biosynthesis of amino acids
	313.61	145.02	C_5_H_6_O_5_	alpha-ketoglutarate	0.11 (0.05)	up	Carbon metabolism
	43.00	163.04	C_9_H_8_O_3_	phenylpyruvate	0.26 (0.14)	up	Phenylalanine metabolism
	238.70	166.08	C_9_H_11_NO_2_	L-phenylalanine	0.20 (0.07)	up	Phenylalanine metabolism


## Discussion

The mutagenesis of the target gene by the CRISPR/Cas9 system-guided usually results in the deletion of the target bases or insertion of a single base in the target fragment, while the insertion mutation is mainly caused by inserting an A or a T base (Zhang H. et al., 2017). In our study, with a help of the CRISPR/Cas9 technology, the bases deletion in the line 9 and an insertion of an A base in the line 13 effectively generated the mutants with the mutagenesis of the *OsPPa6* gene. Highly homology between the OsPPa6 protein and the sPPases in *Z. mays, T. halophila*, and *A. thaliana* seems to indicate a possible regulatory role of the *OsPPa6* gene in response to abiotic stresses. As previously reported, the gene encoding the sPPase is induced by salt stress and involved in energy metabolism in young leaf tissues of the *Z. mays*, and maize growing cells has the cell-age specificity in response to salinity stress (Kravchik and Bernstein, 2013). He et al. (2017) found that the *ThPP1* gene in *T. halophila* is induced by AS and confers an enhanced the alkaline tolerance in rice. In *Arabidopsis*, the AtPPa6 is an exclusively chloroplastic enzyme, and truly essential for plant survival under abiotic stress (Gutiérrez-Luna et al., 2018). Similarly, the up-regulation of the *OsPPa6* gene transcripts might play an important regulatory role in enhancing alkaline tolerance in rice.

Not only responding to abiotic stresses, the sPPases also exert an influence on the plant phenotype. de Graaf et al. (2006) believed that a lower sPPase activity severely hinders the growth of pollen tube in the *Papaver*, and silencing the gene encoding sPPase displays a stunting and bushiness phenotype of potato (Eamens et al., 2014). Our finding basically suggests that the delayed growth of rice mutant obviously is associated with the loss of the activity of the OsPPa6 protein, and led to decreases in both biomass and grain yield, indicating the loss of function of the *OsPPa6* gene, and thereby increased the accumulation of excess PPi in plant cells. However, Jelitto et al. (1992) tentatively transformed the *E. coli* pyrophosphatase gene into potato and tobacco, and observed an apparent growth inhibition of the transgenic plants accompanying an occurrence of yellow leaves. Accordingly, the inhibition or promotion in the sPPase activity would change the PPi/Pi ratio in plant cells and affect the plant growth. Therefore, maintaining PPi/Pi equilibrium is essential for plant’s growth and development.

Maintenance of PPi/Pi equilibrium in plant cells is closely with PPi hydrolysis which is essential for maintaining plant growth, and PPi is usually catalyzed to produce Pi by the sPPase, while Pi provides an important substrate for ATP synthesis by promoting ADP phosphorylation in plant metabolism (Pietropaolo et al., 2016). In our study, more accumulation of PPi in rice mutants is associated with the mutagenesis of the *OsPPa6* gene, because the weak activity of the OsPPa6 protein reduced the accumulation of Pi and ATP. Particularly, a lower accumulation of ATP not only interferes in gluconeogenesis, amino acid activation and DNA replication, but also influences the synthesis and conversion of carbohydrate by energy supply in plants under AS (Vanegas et al., 2015). Generally, alteration of PPi metabolism affects the metabolism of carbohydrates, which conversely change the distribution of carbon in both source and sink organs. Report showed that the specific overexpression of a bacterial pyrophosphatase increases the contents of sucrose and glucose in tomato fruit (Osorio et al., 2013). Geigenberger et al. (1996) found that the transgenic tobacco expressing the gene encoding a *E. coli* PPase increases starch accumulation. Study also proved that the changes of the PPi contents in the transgenic potato overexpressing *E. coli* pyrophosphatase gene affects the metabolism of starch and sucrose (Farré et al., 2000). In this study, lower accumulation of sucrose and starch in the mutants might result from the alteration of PPi enrichment and the reduction of ATP energy supply, lowering the conversion efficiency of carbohydrate in rice. Generally, the carbohydrates conversion is controlled by the photosynthetic process, while both the efficiency of photosynthetic electron transport and PSII light capture in photosynthesis are limited by a higher pH, thus directly affecting P_n_ (Wu et al., 2014). The ThPP1 protein could specifically interact with a PSII light-harvesting-Chl-binding protein, and the transgenic rice overexpressing the *ThPP1* gene encoding the sPPase increased the P_n_ and the accumulation of chlorophyll under AS (He et al., 2017). This study gave a fact that the decreases in the photosynthesis and the chlorophyll contents in the mutants has a closely association with the mutagenesis of the *OsPPa6* gene because of shortage of energy supply of the ATP-mediated, thus lowering the adaptability of rice mutant to high alkalinity. In photosynthesis, photorespiration is an important regulatory factor participating in the re-assimilation of ammonia, and directly affects nitrogen assimilation that is usually controlled by GS2 in plants, and the transgenic rice overexpressing the *OsGS2* gene enhanced the photosynthetic intensity and reduced the photorespiration, and conferred an enhanced salt tolerance (Hoshida et al., 2000), indicating that an enhanced increases in the GS activity is beneficial to the reuse of nitrogen in plants, thus improving plant salt tolerance. In this study, the lower transcripts of the *OsGS2* gene in the mutants played a negative regulatory role in improving rice alkali tolerance because excess Na^+^ accumulation from AS severely affected the photosynthesis in rice.

Both soluble sugar and proline play important regulatory roles in the osmotic regulation of cells and alleviating the alkaline damage to the plants (Székely et al., 2008). Study also confirmed that the mutants *Lotus japonicus* lacking GS2 activity lower proline accumulation, and exhibit more sensitive to drought stress (Díaz et al., 2010). Our data also demonstrate a closely association between the OsGS2 activity and proline accumulation in rice in response to AS, and both soluble sugar and MDA exhibit the differentially accumulated responses to the abiotic stresses, and leading to the changes of osmotic potentials in plant cells (Mittler, 2002; Lv et al., 2013). Both the transgenic *Eleusine coracana* and Brahmi overexpressing a *SbVPPase* gene, encodes a vacuolar proton pyrophosphatase in *Sorghum bicolor*, increases the accumulation of proline and soluble sugar, but reduces the MDA contents under salt stress (Anjaneyulu et al., 2014; Ahire et al., 2018). In our study, the mutagenesis of the *OsPPa6* gene might be an important modulator lowering the contents of soluble sugar and proline, and increasing osmotic potentials and MDA accumulation in the mutants because of the activity changes of the sPPase under AS, indicating that the loss of function of the *OsPPa6* gene affected the accumulation of compatible solutes in rice and exerted negative effects on the maintenance of the osmotic equilibrium in plant cells, and aggravated the alkali damage to the plant cell membranes. The osmotic equilibrium in leaves is closely associated with the accumulation of the Na^+^ and K^+^ under abiotic stresses, and directly affects the plant tolerance to the AS (Yang et al., 2008; Chen et al., 2011). The changes of osmotic potentials in leaves are generally related to the intracellular ion homeostasis which is usually reflected by the acquisition and distribution of K^+^ and Na^+^, since the accumulation of K^+^ has a balance role in lowering the toxicity damage of excess Na^+^ (Deinlein et al., 2014). The homoeostasis of Na^+^ and K^+^ in plant cells is usually controlled by the H^+^-PPase. Lv et al. (2015) reported that the *Arabidopsis* overexpressing the *SeVP1* and *SeVP2* gene increased K^+^ accumulation, but decreased Na^+^ contents, maintaining higher K^+^/Na^+^ ratio in leaves under salt and low nitrogen stresses. Our measurements indicate that like the H^+^-PPase, the *OsPPa6* gene encoding a sPPase might have similar regulatory role in the acquisition and transport of K^+^ and Na^+^, because the mutagenesis of the *OsPPa6* gene significantly exhibited a negative regulatory role in maintaining homoeostasis of Na^+^ and K^+^ in the mutants under AS, thereby lowering the transport ability and compartmentation of Na^+^, increasing the accumulation of excess Na^+^ in plant cells.

The intracellular imbalance of Na^+^ and K^+^ usually changes the distribution of metabolites and disturbs the metabolism in plants (Wu, 2018). In this work, the differential metabolites between the mutant and the WT show that the mutagenesis of the *OsPPa6* gene altered accumulation profiles of these metabolites in rice (Table 5). Reportedly, both phosphorylcholine and choline are two components of cell phospholipids, and mainly involved in the glycerophospholipid metabolism pathway by maintaining the cell’s structure and physiological function in plants, and confer a regulation of the osmotic pressure (Devaux, 1991). In our measurement, the obvious down-regulations of the phosphorylcholine and choline in the mutant imply that increases of osmotic potentials triggered Na^+^ ion damage to the cell membrane under AS. Except the phosphorylcholine and choline, as an important primary metabolite, anthranilic acid participates in the biosynthesis of tryptophan in plants. Jing et al. (2018) reported that the optimized anthranilic acid feeding to the engineered *E. coli* strain would improve L-tryptophan production, while tryptophan not only involves in the protein synthesis, but also is an important precursor substance for biosynthesis of auxin and alkaloid in diverse plants (Hibino and Choshi, 2001). In this study, a lower accumulation of anthranilic acid in the mutant might reduce the tryptophan contents, thus affecting rice growth and development. As an important bioactive plant flavonoid, apigenin is mainly involved in the flavone and flavonol biosynthesis pathway, and increases the resistance of the fungal and oomycetes pathogens in soybean (Jiang et al., 2012), and reduces the damage to the biomembrane by scavenging lipid peroxide, NO free radical, oxygen free radical, and superoxide anion radical (Husain et al., 1987). In this study, little accumulation of the apigenin in the mutant suggests that the mutagenesis of the *OsPPa6* gene increased the harmful free radicals in the cell membrane, which is also suffered from the more damage of MDA. In higher plants, the biosynthesis process of lignin is elucidated by coniferol (Boerjan et al., 2003). Hänninen et al. (2011) found that the amounts of lignin in two similar wood species are dependent on the accumulation profiles of coniferyl alcohol. Also, the lignin plays an important regulatory role in enhancing the mechanical strength of cells and tissues under abiotic stresses (Taylor-Teeples et al., 2015). Our data indicates that the mutagenesis of the *OsPPa6* gene weaken the lignin synthesis and cells mechanical strength, leading to the down-regulation of the coniferol contents in the mutant under AS. Meanwhile, the dodecanoic acid from secondary metabolism in plant also functions in improving the stress resistance through a pathway of synergistic reaction (Peñuelas et al., 1996; López-Villalobos et al., 2011). Our study reveals that lower alkaline tolerance in the mutant might have an association with the reduction of dodecanoic acid. While L-valine has a feedback inhibitory role on the acetolactate synthase (ALS) catalyzing the first step in the biosynthesis of valine, leucine, and isoleucine (Pang and Duggleby, 1999). Our data demonstrate that a lower ALS activity possibly inhibits the synthesis of these amino acids, and thereby affected the accumulation of related protein in the mutant. Ferrario-Méry et al. (2001) reported that both the α-ketoglutarate and glutamine are two metabolite signal molecules participating in ammonia assimilation and transcripts of the gene encoding a nitrate reductase in the transgenic tobacco, and the negative effect of glutamine on the abundance of the nitrate reductase was offset by highly enrichment of α-ketoglutarate mainly deriving from respiration, thus affecting the carbon-nitrogen metabolism in plant. In this study, the up-regulation of the α-ketoglutarate in the mutant is likely associated with the increases of respiration rate in rice at the beginning of AS. Study confirmed that the phenylpropanoid synthesis is an important metabolism pathway in plants, and all substances containing phenylpropane skeleton such as phenolic compounds, alkaloids and flavonoids, are almost produced directly or indirectly by a catalyzation reaction of phenylalanine ammonialyase (PAL) that links primary and secondary metabolism (Jones, 1984). In present study, more accumulation of phenylpyruvate and L-phenylalanine in the mutant might be caused by a lower PAL activity, thus influencing the synthesis of secondary metabolites in rice.

Collectively, our study shows that the mutagenesis of the *OsPPa6* gene encoding a sPPase obviously reveals a lower alkaline tolerance in rice although more detailed and in depth analyses are required. This study provides an inspiration that it would be interesting to investigate whether both the *OsPPa6* itself and its homologous confers a regulatory role in improving rice tolerance in response to the other abiotic stresses such as high salinity and drought, and better utilize the stress-resistant genes for the cultivation of stress tolerant crops.

## Conclusion

In summary, the *OsPPa6* gene is an important regulatory factor conferring an adaptive acclimation of alkaline tolerance in rice. The mutants with the mutagenesis of the *OsPPa6* gene not only showed dwarf phenotype, but also triggered a series of adverse physiological changes, thus resulting in a lower biomass, especially under AS. The mutagenesis of the *OsPPa6* gene led to an imbalance of osmotic potentials and disorder of physiological metabolites in plant cells under AS, indicating that when the *OsPPa6* gene normally functions, a beneficial alteration of physiological metabolites positively contribute to an enhanced increase of alkali tolerance in rice. This study provides an insight into the regulatory role of the *OsPPa6* gene encoding a soluble inorganic pyrophosphatase in adaptive acclimation of plants to AS.

## Data Availability

All datasets generated for this study are included in the manuscript and/or the Supplementary Files.

## Author Contributions

BW performed entire experiment and the preliminary manuscript. GX, ZL, RH, JH, and SH participated in the physiological measurements. LL and XC designed the experiment. XC corrected the manuscript.

## Conflict of Interest Statement

The authors declare that the research was conducted in the absence of any commercial or financial relationships that could be construed as a potential conflict of interest.

## Footnotes

1 http://www.rgenome.net/cas-offinder/

2 http://crispr.mit.edu

3 http://www.ricedata.cn
