# Supplementary material for: Mutagenesis Reveals That the OsPPa6 Gene Is Required for Enhancing the Alkaline Tolerance in Rice
Source: Front Plant Sci. 2019 Jun 11;10:759. doi: 10.3389/fpls.2019.00759 (PMC6580931; doi:10.3389/fpls.2019.00759)
Supplement: Supplementary file 1 [file Data_Sheet_1.pdf]

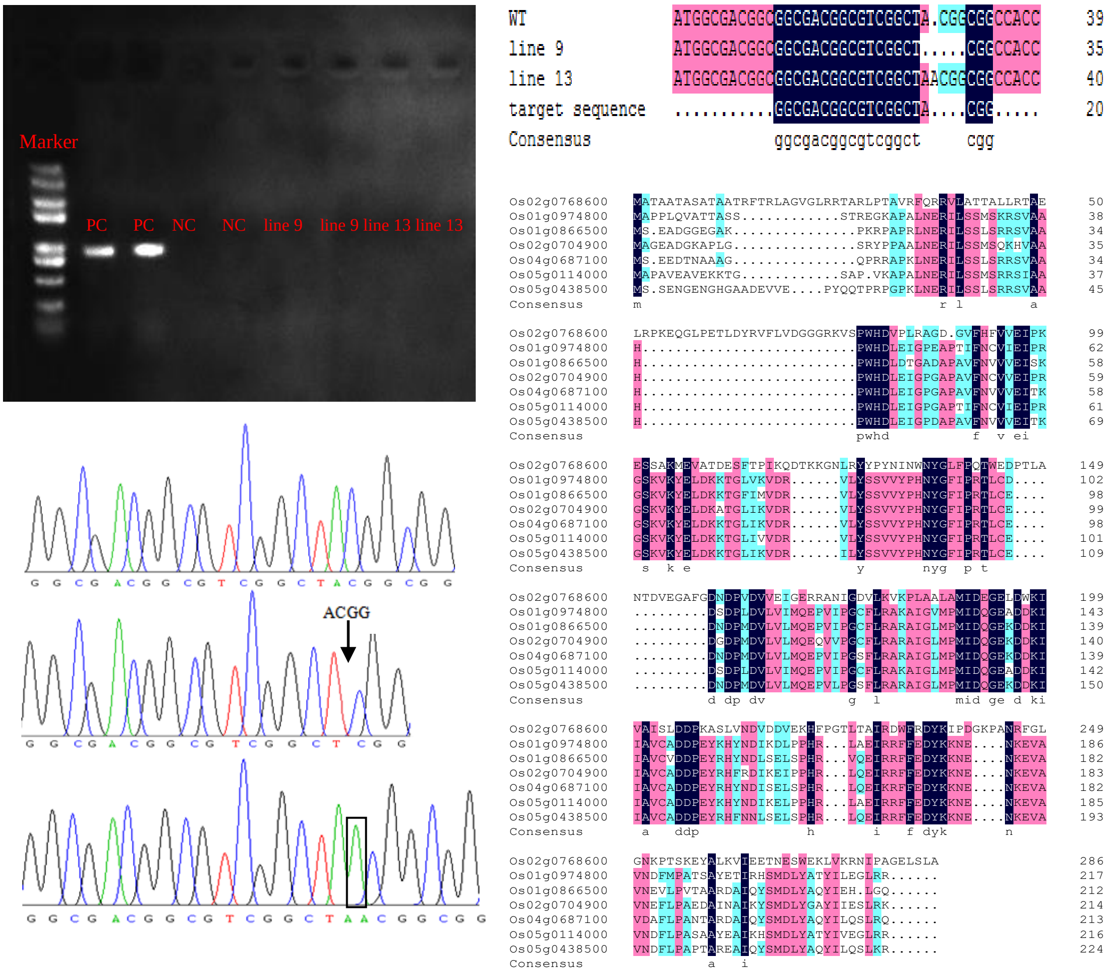

Fig. S1 PCR detection of the mutants with cas9-free and bioinformatic analyses of the *OsPPa6* gene and putative protein sequences of sPPase in rice **(a)**Electrophoresis map from PCR product. "PC" represents the positive control, "NC" represents the negative control; "9" represents the mutant line 9; "13" represents the mutant line 13; **(b)** Nucleotide sequence peak map of *OsPPa6* in the mutants and wild type; **(c)** Multiple alignments of the seven putative protein sequences of the sPPases in rice. Os02g0768600 (accession: BAS81093); Os01g0974800 (accession:BAS76458); Os01g0866500 (accession: BAS75387); Os02g0704900 (accession:BAS80505); Os04g0687100 (accession: BAS91745); Os05g0114000 (accession: BAS91951); Os05g0438500 (accession: BAS94221).

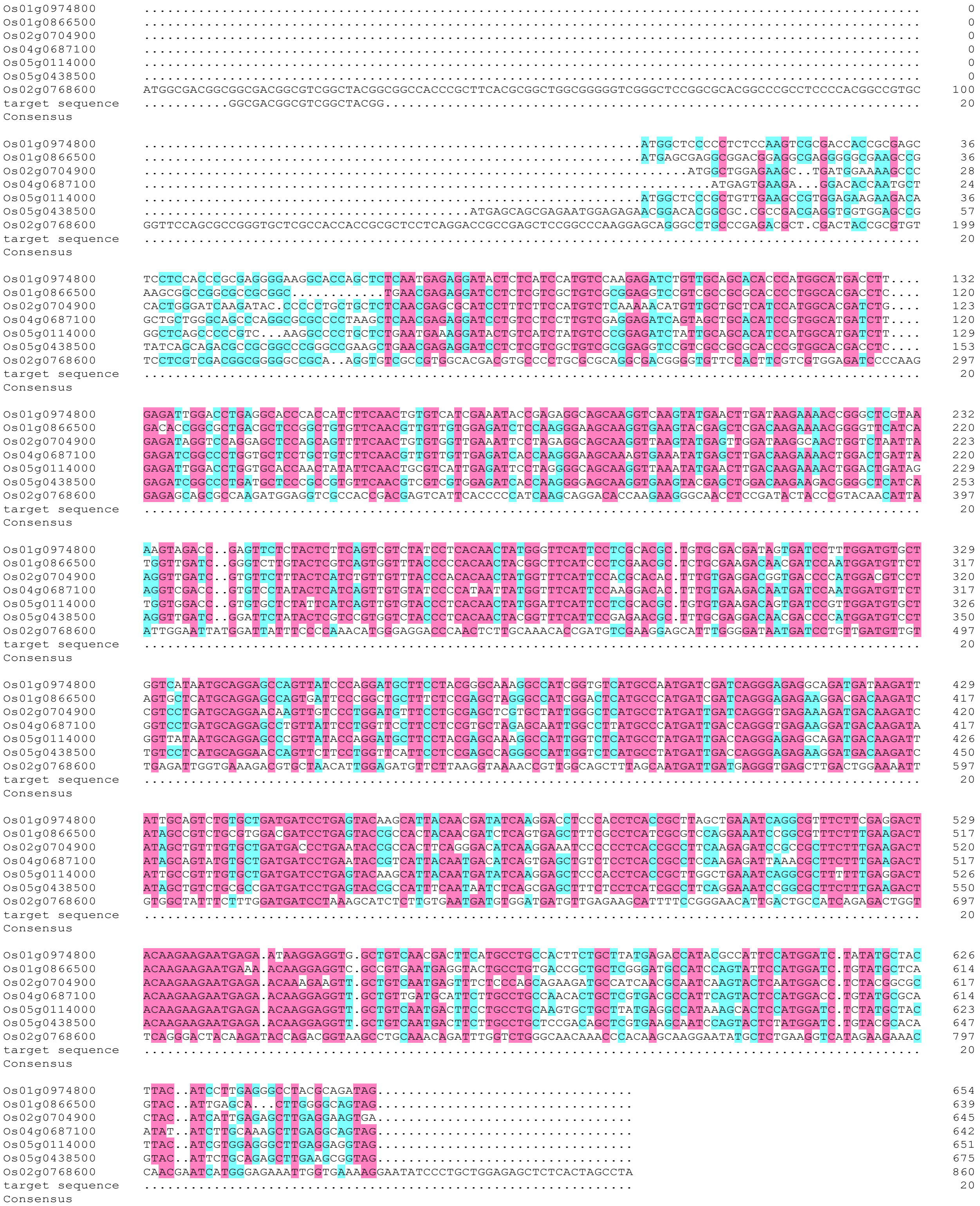

Fig. S2 Nucleotide sequences alignmentof the seven putative gene encoding sPPase in rice.

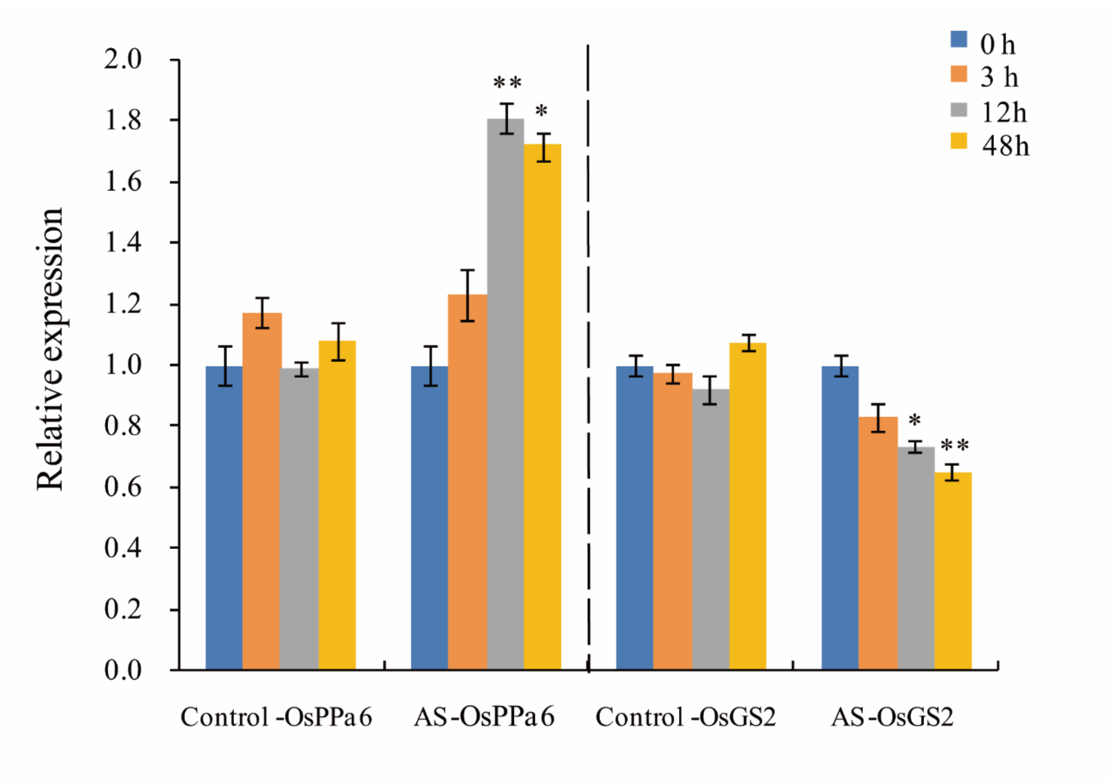

Fig. S3 Relative expression levels of the *OsPPa6* gene and the *OsGS2* gene in shoots of the wild type. Values are mean±SD of three replications and asterisks denote Student's test significance at level of P ≤0.05 (*) or P ≤0.01 (**).

Table S1 Primers used of PCR and qRT-PCR in this study

Primer name	Primer sequences
sgRNA-F	5’-GGCAGGCGACGGCGTCGGCTACGG-3’
sgRNA-R	5’-AAACCCGTAGCCGACGCCGTCGCC-3’
U3-F	5’-GTCGTTTCCCGCCTTCAGTTTTGCATGCCTGCAGGTCGACG-3’
U3-R	5’-CTGTCAAACACTGATAGTTTGGATCCTCTAGAGATTATG-3’
OsPPa6-F	5’-TCCCACTTTCCAGTCTCC-3’
OsPPa6-R	5’-GTTGCCCTTCTTGGTGTC-3’
Cas9-F	5’-AGAGGACTTCTACCCGTTCC-3’
Cas9-R	5’-CCTTCTGTGTGGTCTGGTTC-3’
qOsPPa6-F	5’-TGAGCTTGACTGGAAAATTGTG-3’
qOsPPa6-R	5’-GCTTCTCAACATCATCCACATC-3’
qOsGS2-F	5’-CAGGCTATATTCAAGGACCCAT-3’
qOsGS2-R	5’- AGCCCTGTTACGTTTGTTAGTA-3’
OsActin1-F	5’-CTAAGCCAAGAGGAGCTGTTAT-3’
OsActin1-R	5’-ATAACAGATAGGCCGGTTGAAA-3’
